# Patients’ experiences with virtual group gut-directed hypnotherapy: A qualitative study

**DOI:** 10.3389/fmed.2023.1066452

**Published:** 2023-02-22

**Authors:** Jessica Gerson, Prianca Tawde, Ghoncheh Ghiasian, Jessica K. Salwen-Deremer

**Affiliations:** ^1^Division of Gastroenterology, Inflammatory Bowel Disease Center, New York University (NYU) Langone Health, New York, NY, United States; ^2^The Geisel School of Medicine at Dartmouth, Lebanon, NH, United States; ^3^Departments of Psychiatry and Medicine, Dartmouth Hitchcock Medical Center, Lebanon, NH, United States

**Keywords:** brain-gut behavior therapy, psychogastroenterology, hypnotherapy, irritable bowel syndrome, telemedicine, group therapy

## Abstract

**Background:**

Hypnotherapy is a useful treatment for a variety of gastrointestinal conditions. While there is strong evidence for delivering other treatments virtually and in groups, there is no research thus far on delivering hypnotherapy in this format. Given the growth of both psychogastroenterology and telehealth, these methods should be explored as they have great potential for increasing access and cost-effectiveness of intervention.

**Aims:**

This qualitative study was developed to help understand patients experiences in virtual, group-based, gut-directed hypnotherapy (GDH) in two different institutions.

**Methods:**

Authors developed a qualitative interview with the assistance of two patient partners and then recruited patients from New York University and Dartmouth Health to participate. Interviews were completed one-on-one with patients who started and then completed GDH (≥5 visits) and who did not complete GDH (≤3 visits). Data were coded and then analyzed using thematic analysis.

**Results:**

Twenty-one patients from NYU and Dartmouth participated in qualitative interviews. Broadly, patients reported coming to GDH because they believed in the importance of the mind-body connection or were desperate for treatment. Regardless of why patients came to GDH, they generally reported positive outcomes for GI symptoms and for other physical and mental health conditions. Most patients appreciated the group and virtual formats, though some concerns about inflexible schedules and lack of anonymity were voiced. Despite these concerns, there was broad support for virtual, group-based GDH and general excitement for behavioral health programming.

**Conclusion:**

Virtual, group-based GDH is an acceptable treatment for patients from rural and urban settings. Given the possible improvements in access and cost-effectiveness that this treatment modality can provide, GI practices may want to consider it in lieu of or in addition to the traditional one-on-one treatment format. Barriers and facilitators and recommendations for practice are discussed.

## Introduction

A number of psychological variables are known to be associated with gastrointestinal (GI) conditions, and often either influence the trajectory of physical symptoms and/or impact their emotional sequelae. For example, people with irritable bowel syndrome (IBS) frequently have premorbid psychiatric conditions and trauma histories, and people with both IBS and inflammatory bowel disease (IBD) often report worsening anxiety and depression as their gastrointestinal symptoms increase in frequency and/or severity (1, 2). There is also extensive research on the bidirectional brain-gut connection. Phenomena such as visceral hypersensitivity, which tends to worsen individuals’ subjective experiences of gut activity, can result from changes in vagal nerve activity and/or over-activation of the sympathetic nervous system. For example, heart rate variability, a physiological marker of stress, is altered in people with IBS and in people with IBD (3, 4). As such, recent guidelines highlight the importance of incorporating GI psychology interventions (termed “brain-gut behavior therapies”) into management of GI conditions (5–7).

One of the longest-standing and most widely empirically investigated brain-gut behavior therapies is gut-directed hypnotherapy (GDH). During GDH, the therapist helps the patient attain a state of deep relaxation through physically releasing tension and visualizing relaxing imagery, and then providing hypnotic “suggestions” (8). While this treatment was originally developed for people with irritable bowel syndrome (IBS), it has since been expanded upon and investigated more widely. Numerous RCTs have indicated that GDH is effective for people with refractory IBS and recent research suggests it has the potential to increase time spent in remission in people with ulcerative colitis (9, 10). Hypnotherapy is generally accepted as a form of therapy by the public, however there are many barriers to care that prevent patients from engaging in treatment, including misconceptions, cost, and time (11).

While there is a growing movement to incorporate GI psychology (also termed psychogastroenterology or GI behavioral health) into GI practices, particularly in academic medical centers, GI psychologists often find themselves inundated with referrals and unable to see all patients in a timely manner. One solution to this access problem involves treating patients in a group format. Not only do groups offer social support, normalization, and better illness management, they can also improve efficiency and cost-effectiveness (12). Recent research supports the delivery of gut-directed hypnotherapy in a group format (13, 14). Additionally, services delivered *via* telehealth are not only as effective as those delivered in person, but also offer a potential solution to access problems (15). Patients who have experienced virtual hypnosis report that they are not only satisfied with the experience, but some express even preferring it to face to face, for reasons including feeling less intimidated (16).

In response to both the rising demand for GDH and the COVID-19 pandemic, both JG and JSD began offering virtual, group-based GDH. To our knowledge, few if any GI psychologists have combined these three factors (gut-directed hypnotherapy, virtual treatment, and group therapy). Thus, we sought to explore the patient experience in these programs and to compare their experiences in our two unique settings to improve the variability in patients’ experiences and generalizability of findings.

## Materials and methods

### Program structure

#### Dartmouth health

Dartmouth-Hitchcock Medical Center is a rural academic medical center. In the Section of Gastroenterology, we see patients from all over Northern New England; Maine, New Hampshire, and Vermont are, respectively, considered the 1st, 11th, and 2nd most rural states in the United States (17). Patients at Dartmouth are enrolled in GDH groups as a cohort. For each cohort, the group meets every other week for a total of seven visits, closely following the North Carolina Protocol (18). Patients do not need to be evaluated by a psychologist prior to joining a group; they are screened for appropriateness by an advanced practice provider, and if they pass the screen they are able to enroll in a group. Groups took place *via* zoom and use synchronous audio/video technology. Patients’ insurance companies were billed for each visit.

#### NYU

NYU is an urban private teaching hospital serving patients from all five boroughs of the New York City Metropolitan area, as well as patients who travel to the center from suburbs, other areas adjacent to the city, and/or the state of New Jersey. NYU utilized a rolling admission system for patient enrollment and patients can attend an unlimited number of visits. A social work student (GG) evaluates patients for appropriateness prior to them accessing GDH visits. Groups took place *via* zoom and use synchronous audio/video technology. Patients were charged a modest fee for each visit and insurance was not billed; for some patients in need the fee was waived. Consistent with the extant literature, many patients who attend NYU have been in therapy previously. Broadly, although the prevalence of psychiatric disorders is similar between US adults living in rural and urban areas, adults residing in rural areas receive mental health treatment less frequently due to reduced access to providers and limited availability of specialty care (19).

#### Participant selection

Authors JG and JS-D contacted patients who participated in a virtual group GDH program in the past 6 months. Applicable patients had to have attended between 1 and 3 group visits (considered *non-completers*) or 5 or more visits (considered *completers*).

### Data collection

#### Interview guide

Authors JG and JS-D developed the initial interview guide. All authors discussed this initial draft and then collaboratively refined the guide. The interview guide was then pilot tested with two patients, one of whom had experience in qualitative interviewing. Following this pilot testing, the guide was refined again based on patient feedback. The final interview guide included 23 questions designed to probe for information on reasons for engagement in GDH, treatment outcomes, treatment experience, and barriers and facilitators to engagement (see Supplementary Materials for full interview guide). Participants were also asked whether they would choose individual vs. group format or in-person vs. virtual if they were to repeat their GDH experience.

#### Interview format

Interviews were conducted one-to-one *via* telephone or audio only teleconferencing (based on participant preference) at a single point in time. Only the interviewer and participant were present during the interview. Participants could be at a location of their choosing during the interview, and most elected to participate from home. Interview duration ranged from 10 to 30 min (mode = ∼30 min) during which the interviewer took extensive field notes. Any points of clarification were addressed after the interview using audio recordings. Transcripts were not returned to participants for comment or correction. Interviewers monitored the extent of data saturation over the course of their interviews.

#### Interviewer characteristics

All interviews were completed by GG or PT, both of whom identify as a woman. Neither interviewer had any relationships with the participants prior to this study. At the time of data collection, GG held a Masters in Public Health, served as a research program manager for the NYU IBD Center, and was a student in a Masters of Social Work program. PT was a 4th year medical student who has been involved in gastroenterology research through Dartmouth-Hitchcock for several years. Both GG and PT had experience in clinical interviewing through their individual programs of study. GG completed qualitative interview training as a part of her graduate studies in Public Health and PT completed qualitative interview training as part of her research experiences with previous academic projects. Both received training collaboratively from JG and JSD to ensure consistency in training background and interview formats. Participants were informed that their interviewer was a [social work/medical] student involved in the GI program at the respective hospital and interested in how GI behavioral health can improve quality of life in people with GI conditions.

#### Demographic data

Age, gender, self-described ethnicity, medical diagnosis, and psychiatric diagnosis at time of interview were abstracted from participants’ charts (Table 1).

**TABLE 1:** Aggregated participant demographics.

Age	Range: 29 to 77 years
	Mean = 47.7, SD = 16.3
Location	Dartmouth: *n* = 11
	NYU: *n* = 10
Gender	Women: *n* = 16
	Men: 4
	Transgender/gender diverse: *n* = 1
Race/ethnicity	Caucasian/white: 19
	Hispanic: 1
	Preferred not to disclose: 1
Medical diagnoses (note: some participants endorsed multiple primary medical diagnoses following review of diagnoses noted in chart)	Chronic pelvic pain: *n* = 1
	Diverticulosis: *n* = 1
	Dyspepsia: *n* = 1
	Dysphagia: *n* = 1
	Fecal incontinence: *n* = 1
	Functional gastrointestinal disorder: *n* = 5
	Gastritis: *n* = 1
	Gastroesophageal reflux disease: *n* = 1
	Inflammatory bowel disease: *n* = 4
	Irritable bowel syndrome: *n* = 10
Psychiatric diagnoses (note: some participants endorsed multiple primary psychiatric diagnoses following review of diagnoses noted in chart)	Adjustment disorder: *n* = 1
	Anxiety: *n* = 12
	Bipolar disorder: *n* = 1
	Depression: *n* = 6
	Insomnia: *n* = 1
	Posttraumatic stress disorder: *n* = 4
	Obsessive compulsive disorder: *n* = 2
	Schizoaffective disorder: *n* = 1
	None: *n* = 4

### Human subjects

These data were collected as part of our quality improvement initiatives and were determined to be exempt from IRB review by both Dartmouth Health and NYU. All patients verbally consented to participate in the interviews.

### Data analysis

Interviews were conducted independently at each site until the interviewer felt certain that thematic saturation had been reached. While saturation is a widely debated topic, we defined thematic saturation based on two points: first, the point at which interview content became redundant as determined by the interviewers, and second, the time during the coding process when no new codes were established with further interview content (20, 21). Qualitative interview analysis was completed using ATLAS.ti version 22.0.2. Initially, two interviews were coded by two coders (TD and JS-D). The subsequent interviews were analyzed by an individual coder (PT). After the initial coding was complete, JSD reviewed 20% of interviews, stratified by site and GDH completion, and applied her own codes to align on reproducibility. PT and JSD then collaboratively reviewed codes to reduce redundancy. Four major themes were generated inductively, and these themes were reviewed by all four authors. Participants did not provide feedback on the themes derived from the interviews.

## Results

**FIGURE 1:**
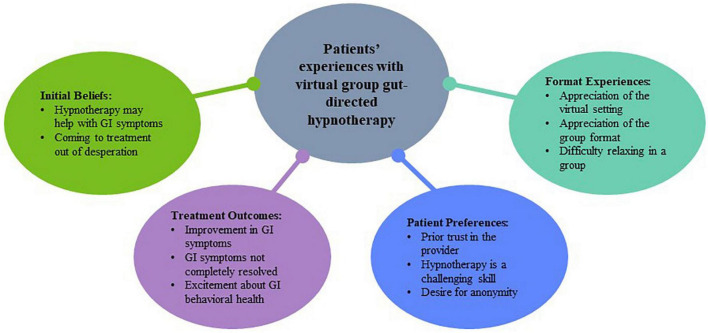
Consolidated themes describing patient experience with virtual group gut-directed.

At Dartmouth Health, 19 patients were approached and 11 patients agreed to participate. At NYU, 16 patients were approached and 10 patients agreed to participate. Possible participants were approached on a rolling basis, with addition of new participants until interviewers felt certain that thematic saturation had been achieved. A total of 21 participants were included in the final sample, including 10 from NYU and 11 from Dartmouth Health. During the interview coding process, thematic saturation was reached after 6 NYU interviews were coded and after 9 Dartmouth Health interviews were coded. A consolidation of the themes describing patient experiences can be found in Figure 1. Participants ranged in age from 29 to 77 years (Mean = 47.7, SD = 16.3), 76% (*n* = 16) were female, and 90.5% (*n* = 19) were White. The referral to GDH was predominantly made for a disorder of brain-gut interaction (*n* = 17; 81%). Many had a comorbid mental health diagnosis (*n* = 17; 81%), most commonly an anxiety disorder (*n* = 12; 57%).

### Initial beliefs

Patients across the two programs came to hypnosis with varying expectations for how behavioral health could address their GI symptoms and experience with behavioral health in general.

#### Beliefs that hypnotherapy could work

A group of patients had positive experiences with behavioral health, recognized a correlation between their gut and mental health, and/or believed that hypnotherapy would work well for managing or reducing their GI symptoms.

• *The worse you feel mentally the worse you feel physically. It all takes over.—Patient 6*
• *I believed that [hypnotherapy] could be helpful and successful.—Patient 4*
• *Cognitive Behavioral Therapy opened my mind to the gut-brain axis.—Patient 10*

#### Coming to hypnotherapy out of desperation

While some came to hypnotherapy with an understanding of the gut and brain connection, others were unaware there was a connection and did not believe this treatment would work. Some patients even indicated that they were “insulted” by the implication their brain was involved. However, across individuals who indicated they came out of desperation, they found the education on the gut/brain axis to be enlightening and the hypnotherapy experience to be helpful. Patients ended up trying group based hypnotherapy because they felt frustrated with or hopeless about their therapeutic options. Across completers and non-completers of the program, patients saw the value of the program. Further, all patients interviewed indicated they would recommend the program to others, irrespective of completion status.

• *I was a bit insulted and didn’t understand the connection and I thought wait, my symptoms are real. Then when I started, I started to understand why I needed this so badly.—Patient 18*
• *Hypnotherapy was a new thing. Born out of desperation related to my symptoms.—Patient 11*
• *I’ve suffered from reflux for 25 years and I have a small hiatal hernia. When I asked how its treated, she [the GI provider] said PPIs don’t work and of course I had been on them for years and I knew it didn’t work. So she said we use a low dose antidepressant, and I said what else can I do and she said hypnotherapy!—Patient 15*

### Outcomes

Many patients indicated that they saw improvement or stability of their symptoms through this intervention.

#### Improvement in both GI symptoms and related experiences

Patients saw direct improvement in GI symptoms and reduction in the vicious cycle wherein GI symptoms caused emotional distress, leading to more GI symptoms, more distress, etc., once their symptoms flared. Patients also reported improvement in anxiety and increased in their ability to engage with relaxing sensations with their practice of hypnotherapy. This improvement in anxiety and relaxation attributed to the program was seen even in patients that did not complete the program.

• *Yes, I find myself doing that sensory exercise when I am overwhelmed with appointments or in the hospital. I use that to calm myself down which helps me feel physically well.—*Patient 6
• *My gastrointestinal pain has improved.—Patient 4*
• *I think at that time I felt helpless so having this [the GDH program] helped reduce my anxiety.—Patient 17*

#### GI issues were not completely resolved

While many patients experienced a benefit from hypnotherapy, it was often not able to completely resolve their symptoms. In these cases, patients indicated that the hypnotherapy worked hand in hand with other treatments to improve symptoms.

• *I still have GI problems, but not all of it is psych related and won’t be fixed completely with hypnotherapy.—Patient 11*
• *GI wise, I do have pain issues, gastro pain that I didn’t mention before and they don’t know where that is from and I am still getting the pain but not as often as I was getting them.—Patient 13*

#### Excitement about GI behavioral health in general

Given the benefits from hypnotherapy, many patients were enthusiastic about more programming around group-based hypnotherapy and/or other GI behavioral health interventions.

• *I don’t think hypnotherapy is used enough—Patient 16*
• *I am very grateful and thankful that I had this opportunity to be a part of this experience and it has helped me cope with a lot of my stomach issues and finding coping mechanisms that work.—Patient 18*
• *I love that it is happening. to try to make it more readily available—Patient 2*

### Patient preferences with hypnotherapy

There were several takeaways that emerged across participants who did and did not complete the program and across sites. These takeaways are worth highlighting, as they may impact how we and others shape future programs.

#### Trust in the provider

One key area that participants commented on was the importance of trust in their provider throughout the process. Notably, patients who did and did not know their group leader before joining the group indicated that trusting the provider made a difference in their program engagement and their belief in the potential of the intervention.

• *I think [GI psychologist] is so great at her job*… *I feel like I was a complete mess. I was not in a good place. She is so patient. great answers for everything and she really tries to understand each person.—Patient 6*
• *I think it was good that I had known [GI psychologist] before. Felt comfortable and trusted her. – Patient 11*
• *It would have been helpful to know [GI psychologist] in person before because she is a stranger—Patient 7*

#### Learning hypnotherapy skills is difficult

Another common comment across participants related to how challenging hypnotherapy can be. For some, this difficulty was related to their desire for perfection in their practice, which may not be achievable. For others, this difficulty resulted in struggling with GDH, which paradoxically increased their anxiety. Finally, patients found their homes often had distractions (e.g., children, pets, external sounds) that impacted their ability to fully engage in the hypnotherapy.

• *I don’t know if the sessions work for me. I have learned through the group setting and through the recording but don’t know if I reach the same level as others.—Patient 3*
• *Memory is hard and remembering what to do [during hypnotherapy] is difficult—Patient 11*
• *Family was loud in the next room*.*—Patient 7*

#### Desired anonymity

Participants from both sites desired anonymity and struggled with the hospital-driven protocols that prevented it. Of note, difficulty with the groups not being completely anonymous was a more common complaint in participants who did not complete the programs.

• *I am also not really a fan of being on camera*…*I think if there was [also] a way to block out an individual name. I saw my full name pop up.—Patient 1*

### Patient experiences with format

#### Virtual vs. in-person format

Overall, across completers and non-completers of the program, a majority of the participants appreciated the virtual setting. Irrelevant to urban or rural environments, transportation to the respective hospital/clinic for therapy was described as difficult and time consuming. Participants also endorsed travel time and transportation as a major barrier to other interventions related to their GI pathology. Especially for participants dealing with diarrhea, urgency, and/or incontinence, being in their homes and having easy access to a bathroom was critical to feeling comfortable.

• *“I couldn’t do it virtually I wouldn’t have been able to do it”—Patient 7*
• *“I feel like when you are doing it in person, well I would need an hour to commute and I would not be able to do it midday.”—Patient 16*

For people who did not like the virtual format, they recognized the convenience, but also valued face-to-face care. Notably though, participants who would have preferred in-person care also shared that they likely would not have been able to participate that way.

• *“It helps to be remote because I need to be near a bathroom, but in person would resonate better with me.”—Patient 6*

#### Individual vs. group format

In terms of a group experience, most participants appreciated hearing the sentiments of their group members. They also valued the community and reduced isolation accompanied with a group therapy.

• *It was nice to hear other people’s perspectives—Patient 4*
• *The group setting is nice because I don’t feel alone—Patient 14*

Some detractors of the group experience included an inability to relax or feel vulnerable in front of peers. Patients who struggled with the group setting also endorsed difficulty with the inflexibility of group sessions. While many patients reported desiring individual sessions, most still found the utility of groups especially when it came to access to treatment and hearing group perspectives.

• *I feel like I can be vulnerable enough—Patient 5*
• *People say ‘that they are bleeding’ and these are triggering and strong words to use before a hypnotherapy session, it was triggering—Patient 16*
• *I feel that some of it was that I had so much of my own stuff to deal with that listening to other people’s stuff is not my forte.—Patient 7*
• *Only downfall is the progression over time. You don’t go back and do what you miss. Individual doesn’t advance without you.—Patient 8*
• *I planned on doing more but then I ended up getting a new job and the times were not when I was available. – Patient 7*

Finally, to further understand individuals preferred formats for GDH, they were asked if they could have a “do over,” what format would they have chosen for their treatment. Overall, 70% of responding patients (*n* = 14) reported desiring a virtual environment. With regard to in-person vs. group delivery, 50% of responding patients (*n* = 10) reported valuing a group format.

## Discussion

The purpose of this qualitative study was to better understand patients’ experiences in virtual group GDH programs, comparing patients who did and did not complete group visits and who lived in urban or rural settings. Qualitative interviews revealed key themes related to engagement in GDH, experiences during the program, and key take-aways for others interested in creating similar programs. Broadly, our interviews revealed that patients who did and did not complete virtual group GDH programs and who enrolled through rural or urban programs had similar expectations and experiences. Primarily, interviews revealed two main reasons for enrolling in GDH treatment; some patients entered treatment with strong beliefs in the mind-body connection, and others were either initially ignorant or skeptical about the relationship between the brain and the gut and were driven by desperation. Despite this initial skepticism, most patients ultimately felt positive about the treatment, suggesting that prior beliefs regarding hypnotherapy are not necessarily indicative of outcomes. At the same time, it is also possible that for patients who do not understand the brain-gut connection, they will only consider brain-gut behavior therapies once they become desperate. Thus, there is likely an opportunity for GI providers to deliver education about the brain-gut connection early in treatment and encourage patients to consider brain-gut behavior therapies (5).

With regard to outcomes, as noted, many patients indicated that their GI symptoms or other physical or mental health conditions had improved. Interestingly, this experience was reported in patients who did and did not complete GDH programs. Thus, it is possible that patients who can only commit to a few visits should still be encouraged to participate, particularly if they are able to engage in at-home practice. Importantly, because GDH does not alleviate all symptoms, medical practitioners should make it clear that they will continue to provide follow up care as the patient participates in other interventions. Further, patients’ enthusiasm for GI behavioral health as a practice reflects the importance of supporting the development of robust, comprehensive, and multi/interdisciplinary programs for the care of these patients.

Within the virtual, group-based GDH program, some specific preferences emerged. Patients highlighted the value of having some interaction with the hypnotherapy group leader before starting treatment. In both the Dartmouth and NYU programs, the therapists relied on other clinical staff to screen patients, but perhaps offering a brief conversation with prospective patients prior to starting a group would be helpful for attendance and/or outcomes. Most patients commented on the difficulty of learning self-hypnosis, which highlights the need for group leaders to be aware of and discuss the challenges of individual practice. Other barriers to engagement, including distractions in the home environment and concerns about anonymity, are also important to discuss both early on and throughout the group so that patients feel supported in these areas.

Consistent with the extant literature, patients’ experiences with the delivery format were generally positive. Most patients appreciated being able to complete the treatment virtually as well as in groups, for logistical reasons (e.g., travel, illness), easier access to treatment, and for normalization of their experiences. However, some patients found it harder to relax around others, struggled with rigid scheduling, and/or found others’ experiences to be triggering. Ways to address these issues should be considered by providers looking to build similar programs, for example having some ground rules and agreements about what patients can discuss in groups and allowing for make-up sessions and/or alternative times.

This qualitative study had both strengths and weaknesses. Primarily, the diversity of experiences, including both completers and non-completers and patients from rural and urban setting improves the generalizability of our findings. However, despite efforts to engage a diverse group of participants, nearly all our participants were Caucasian. Future research investigating the experiences of patients from other racial or ethnic backgrounds will be important for understanding individual experiences. Additionally, while qualitative interviews provide rich narratives and give patients the space and time to share their full individual experiences, they lack empirical data. Research that utilizes mixed qualitative and quantitative methods will be important for further understanding how patients’ experiences and symptom outcomes do or do not differ across treatment modalities. Finally, future research investigating how patients are referred to GDH and the role of medical providers in this process could be particularly helpful in getting patients to this evidence-based treatment sooner.

## Conclusion

Overall, virtual, group-based gut-directed hypnotherapy appears to be a promising and novel approach to working with patients with GI conditions. Not only is it economically and logistically appealing, but clinically patients find it helpful in terms of improving both physical and mental health symptoms. The themes that emerged can be addressed thoughtfully by clinicians and may assist others in implementing similar programs, for example dispelling myths prior to treatment, working out the technical aspects, etc. Future directions include outcome research, provider psychoeducation, as well as expanding the model of virtual groups to other aspects of GI psychological care.

## Data availability statement

The datasets presented in this article are not readily available because the data were generated through interviews as part of a quality improvement project. Requests to access the datasets should be directed to JS-D, jessica.k.salwen-deremer@hitchcock.org.

## Ethics statement

These data were collected as part of our quality improvement initiatives and were determined to be exempt from IRB review by both Dartmouth Health and NYU. Written informed consent for participation was not required for this study in accordance with the national legislation and the institutional requirements.

## Author contributions

JG and JS-D designed the interview guide with input from GG and PT. GG and PT conducted qualitative interviews, PT and JS-D coded interviews, and all authors agreed on themes. JG wrote the initial draft of the introduction and discussion. PT and JS-D wrote the initial draft of the method and results. All authors contributed to important intellectual content to subsequent revisions of the manuscript and agreed on this final version.
