# Supplementary material for: Patients’ experiences with virtual group gut-directed hypnotherapy: A qualitative study
Source: Front Med (Lausanne). 2023 Feb 22;10:1066452. doi: 10.3389/fmed.2023.1066452 (PMC9992176; doi:10.3389/fmed.2023.1066452)
Supplement: Supplementary file 1 [file Data_Sheet_1.PDF]

**Qualitative Interview Guide**

**General hypnosis beliefs**

1. What do you think about behavioral health being part of GI care?
2. What did you think about hypnosis as a form of behavioral health treatment?
    a. *Probe for opinions, values*
3. How would you describe the experience of hypnosis to someone who hasn't done it before?

**Treatment modality**

4. What was it like to get hypnosis in a group versus individually?
    a. *Probe for likes, dislikes, pros/cons of being around other people (stigma, support)*
5. What was it like for you to get treatment without being face-to-face?

**Treatment adherence**

6. How many visits did you attend?
7. What were some of the things that made it easier for you to participate in the groups?
    a. *Probe for facilitators (e.g., telehealth, access via Epic online system)*
8. What were some of the things that got in the way of participating or made it difficult?
    b. *Probe for barriers (e.g. telehealth, social determinants of health)*
9. How often did you engage in self-hypnosis practice?
    a. *Probe for barrier/facilitators to practice*

**Treatment outcomes**

10. What about your GI symptoms changed over the course of hypnosis?
11. What changed about other mental health or physical health symptoms as a result of or during hypnosis?
12. What changed about your ability to cope with your GI symptoms?

**Treatment experience**

13. What would you change, if anything, about the treatment overall?
    a. *Probe for frequency, structure, content, etc.*
14. What about the hypnosis was most helpful for you?
15. What part of the hypnosis did you find least helpful?
16. Before we finish up, is there anything else you would like to share about your views, opinions, or experiences in this treatment?
